# Sofosbuvir and ribavirin in acute hepatitis C–infected patient with decompensated cirrhosis

**DOI:** 10.1097/MD.0000000000005555

**Published:** 2016-12-09

**Authors:** Hui Liu, Tong Zhang, Yan Yan

**Affiliations:** Department of Gastroenterology, Second Affiliated Hospital of Dalian Medical University, Dalian, Liaoning Province, China.

**Keywords:** case report, decompensated cirrhosis, HCV, hepatitis, ribavirin, sofosbuvir

## Abstract

**Background:**

The treatment of chronic hepatitis C virus infection has been revolutionized by the advent of direct-acting antiviral agents. However, evidence of its effects on patients with acute hepatitis C (AHC) virus infection is limited.

**Case summary:**

We report the case of a patient with decompensated cirrhosis induced by autoimmune liver disease, whose condition rapidly deteriorated following AHC virus infection. The patient received sofosbuvir and ribavirin combination treatment for 12 weeks. Serum hepatitis C virus RNA remained undetectable 36 weeks after discontinuing sofosbuvir and ribavirin.

**Conclusion:**

Our findings support the use of sofosbuvir and ribavirin as a treatment in AHC patients with decompensated cirrhosis.

## 1 Introduction

The use of direct-acting antiviral (DAA) agents in the treatment of chronic hepatitis C has revolutionized the management and control of this important liver disease.^[1]^ Currently approved DAA therapy with sofosbuvir (SOF)-containing regimens has dramatically improved rates of sustained virological response and shortened treatment duration.^[1]^ In recent publications, response rates of 85% to 90% have been reported in patients with decompensated chronic hepatitis C (Child–Pugh B or C).^[2–4]^ However, DAA agents remain to be prescribed only for chronic hepatitis C virus (HCV) infection. Various studies are underway to assess the use of interferon (IFN)-free DAA combinations in the treatment of acute hepatitis C (AHC) virus monoinfection and coinfection. In most HIV–HCV–coinfected patients with AHC, HCV eradication is achieved with different DAA combinations.^[5]^ We present the case of a patient with AHC with decompensated cirrhosis induced by autoimmune liver disease, who was successfully treated with SOF and ribavirin (RBV).

## 2 Case report

**Figure 1:**
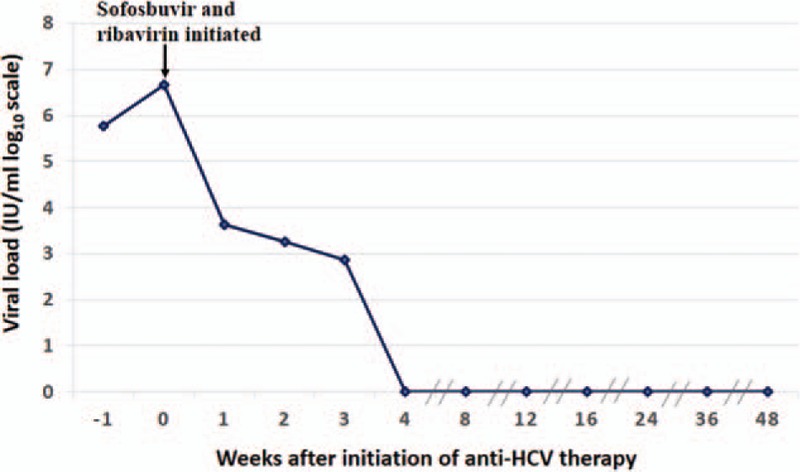
The HCV viral load subsequently decreased after treatment with sofosbuvir and ribavirin. At the beginning of sofosbuvir and ribavirin treatment, HCV RNA viral load was 4.8 × 10^6^ IU/mL. At week 1, the HCV RNA viral load decreased to 4330 IU/mL; at week 2, 1790 IU/mL; and at week 3, 750 IU/mL. At weeks 4, 8, 12, 16, 24, 36, and 48 (posttreatment week 36), HCV viral load was undetectable. HCV = hepatitis C virus.

**Figure 2:**
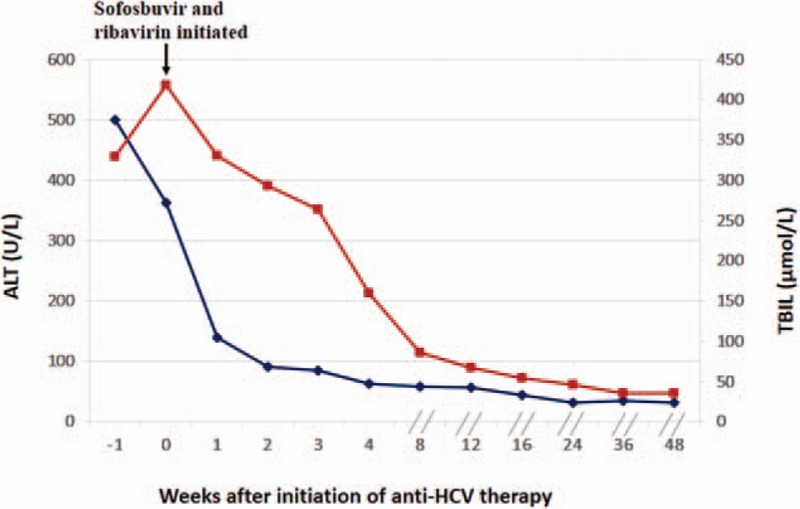
Liver function gradually recovered after treatment with sofosbuvir and ribavirin. By week 8, ALT and TBIL returned to baseline levels. ALT = alanine aminotransferase, HCV = hepatitis C virus, TBIL = total bilirubin.

A 65-year-old woman had been diagnosed with overlap syndrome (autoimmune hepatitis and primary biliary cirrhosis) for 14 years. Initially, she was prescribed ursodeoxycholic acid and prednisone, and refused immunosuppressive drugs. However, due to the presence of gastric ulcer and economic reasons, she stopped taking these medications. When liver test results were abnormal, she would take compound glycyrrhizin tablets to treat the disease. In September 2013, due to hematemesis and melena, rupture and hemorrhage of the esophagofundal varices was found. Computed tomography revealed cirrhosis of the liver, splenomegaly, and ascites. The disease progressed to Child–Pugh C decompensated cirrhosis. The patient received endoscopic therapy, and began taking prescribed ursodeoxycholic acid 750 mg daily. From September 2013 to August 2015, her alanine aminotransferase (ALT) level fluctuated between 27 and 91 U/L, aspartate aminotransferase between 33 and 94 U/L, γ-glutamyl transpeptidase between 73 and 240 U/L, alkaline phosphatase between 130 and 260 U/L, total bilirubin (TBIL) between 39.3 and 74.05 mol/L, direct bilirubin between 17.6 and 34.24 mol/L, albumin between 30 and 35 g/L, antinuclear antibody (ANA) titer between 1:1000 and 1:3200, and antimitochondrial II antibody (AMA-M2) between (+) and (++). In September 2015, the patient consulted her physician in our department for significant jaundice and weakness. Physical examination revealed yellow pigmentation of the skin and sclerae, positive liver palms, spider angioma on the chest, and positive shifting dullness. Results of liver function tests showed the following: ALT, 499.9 U/L; aspartate aminotransferase, 686.8 U/L; albumin, 27.02 g/L; γ-glutamyl transpeptidase, 87.2 U/L; alkaline phosphatase, 225.6 U/L; TBIL, 329.6 mol/L; direct bilirubin, 272.91 mol/L; cholinesterase, 2156.4 U/L; prothrombin time, 17.7 seconds; percentage prothrombin time activity, 60; and platelet count, 45 × 10^9^/L. The anti-HCV antibody test result was positive and the quantitative HCV RNA test result was 6.1 × 10^5^ IU/mL (genotype 1). The ANA titer was 1:3200, and the AMA-M2 was (+). Epigastric enhanced magnetic resonance imaging identified cirrhosis, splenomegaly, ascites, and collateral circulation in the esophagus, fundus of the stomach, and splenic hilum. Magnetic resonance cholangiopancreatography did not detect any abnormality in the intrahepatic and extrahepatic bile ducts. Initially, the treatment included ursodeoxycholic acid at the previous dosage, glycyrrhizinate, human serum albumin, furosemide, spironolactone, lactulose oral solution, and platelet and clotting factor transfusion. However, after 1 week, weakness and jaundice were exacerbated. Liver function test results were significantly abnormal with a TBIL of 417.6 mol/L, prothrombin time of 23.5 seconds, percentage prothrombin time activity of 46, albumin of 23.1 g/L, cholinesterase of 1789.7 U/L, and platelet count of 14 × 10^9^/L. After ruling out other possible causes of acute hepatitis, we thought that the cause of the acute exacerbation of hepatic damage was AHC infection, as liver function was decreased by HCV activity. After discussing the risks and benefits with the patient, we started her on an IFN-free combination therapy of SOF and weight-based RBV for 12 weeks. Gilead-brand SOF (400 mg/d) was used. SOF cannot be bought in China; the patient acquired the medication through her relative who works abroad. The patient was reviewed at treatment weeks 1, 2, 3, 4, 8, and 12 and at posttreatment weeks 4, 12, 24, and 36. At the beginning of SOF and RBV treatment, the HCV RNA viral load was 4.8 × 10^6^ IU/mL. At week 1, the HCV RNA viral load was reduced to 4330 IU/mL; at week 2, 1790 IU/mL; and at week 3, 750 IU/mL. At weeks 4, 8, 12, 16, 24, 36, and 48 (posttreatment week 36), the HCV viral load was undetectable (Fig. 1). By week 4, the patient's symptoms alleviated. By week 8, liver function returned to baseline levels with TBIL of 85.35 mol/L, prothrombin time of 17.0 seconds, albumin of 29.5 g/L, and platelet count of 53.2 × 10^9^/L (Fig. 2).

## 3 Discussion

There are 6 main genotypes of HCV with different responses to treatment. Genotype 1 (HCV-1) is the most common genotype in the world, as well as in China, accounting for 60% of all cases worldwide.^[6]^ Infection with HCV is usually asymptomatic, with only a minority of patients presenting with symptomatic AHC. Herein, we report a rare case of a patient with decompensated cirrhosis induced by autoimmune liver disease–infected AHC. HCV infection is predominantly transmitted by exposure to blood or body fluids. The incubation period of AHC infection after blood transfusion is 2 to 16 weeks (average, 7 weeks). This patient received red blood cell transfusions on 2 separate occasions (in September 2013 and December 2014) to address her anemia due to digestive tract hemorrhage. However, the patient was tested for anti-HCV antibody and HCV-RNA after the second red blood cell transfusion in December 2014, with negative results for both. The anti-HCV antibody test result was also negative in March 2015 and August 2015. Thus, we thought that AHC was not transmitted by blood transfusion. HCV can also be transmitted through broken skin and mucosa. To our knowledge, the patient did not inject drugs or undergo contaminated medical procedures. No other routes of infection were found. Notably, the patient who was undergoing treatment with phototherapy, compound glycyrrhizin tablets, and vitamins had psoriasis of 22 years with severely broken skin. She had hot-spring baths (with a water temperature of about 45°C) once or twice per week with her friend who had a chronic HCV infection from August 2015 to September 2015. Therefore, we thought that HCV might have been transmitted through broken skin. In this case, when acute exacerbation of hepatic damage occurred, we believed that it was appropriate initially to consider the progression of autoimmune liver disease. From September 2013 to August 2015, the ANA titer fluctuated between 1:1000 and 1:3200, and AMA-M2 fluctuated between (+) and (++). The highest levels of ANA and AMA-M2 were 1:3200 and (++), respectively, but the liver dysfunction was mild throughout therapy, including treatment with ursodeoxycholic acid and glycyrrhizinate. In September 2015, liver function dramatically worsened. The ANA titer was 1:3200 and AMA-M2 was (+), both of which did not exceed the prior level. After comprehensive medicine treatment for 1 week, the patient's condition continued to worsen. Thereafter, the patient began antiviral therapy based on the previously administered medicine with the same dosage. No glucocorticoid or immunosuppressant was used, which is necessary in acute exacerbation of autoimmune liver disease. After antiviral therapy for 1 week, the patient's symptoms and liver function improved. Following the reduction of viral load, liver function correspondingly improved. The clinical course did not match the pathological characteristics of acute exacerbation of autoimmune liver disease. No hepatitis A, B, D, or E virus or HIV infection was found. Other possible causes of acute hepatitis, such as drugs, toxic, alcohol, and infection with other pathogens, were also not found. After ruling out other possible causes of acute hepatitis and observing the therapeutic effect of antiviral medication, we concluded that the primary reason of the dramatic deterioration of liver function involved mass liver cell necrosis caused by AHC infection. By restraining and eradicating the hepatitis virus, DAAs played a key role in the rapid decrease of ALT and TBIL. Other comprehensive pharmacological treatment, including ursodeoxycholic acid at the previous dosage, glycyrrhizinate, and platelet and clotting factor transfusion, took subsidiary function in sustaining the liver cell membrane, promoting biliation and egestion, and preventing bleeding. It should also be noted that spontaneous viral resolution occurs in about 20% to 40% of infected patients. Currently, recommendations regarding when to start antiviral treatment in patients with AHC are conflicting. Some scholars recommend antiviral treatment if accompanied by elevated ALT, regardless of other clinical symptoms.^[7]^ Others suggest monitoring through HCV RNA quantification every 4 weeks, and, if patients display positive HCV RNA for 12 weeks, beginning antiviral therapy.^[8]^ In our patient, liver function, blood coagulation function, and platelet count declined significantly after AHC infection, and, thus, we chose antiviral therapy. When IFN was the main drug used in the treatment of HCV, there was no effective antiviral therapy for AHC patients with decompensated cirrhosis. The discovery and application of DAAs brought hope to the patients who need more choices for the treatment of their condition. Several trials had found that DAAs can effectively eradicate HCV in AHC patients coinfected with HIV. However, evidence remains limited. A potent nucleotide analogue, SOF, inhibits HCV NS5B polymerase with pan-genotypic activity. It was approved by the Food and Drug Administration and the European Medicines Agency in combination with RBV as the first IFN-free treatment option for patients with chronic HCV with and without contraindications to pegylated-IFN. The patient exhibited a rapid virological response in 4 weeks, with undetectable amounts of HCV RNA at 36 weeks after SOF and RBV treatment completion. Except autoimmune liver disease and psoriasis, this patient had a history of hypertension for 10 years. Over the course of therapy, the patient's blood pressure dropped from (140–150)/(80–90) to (100–105)/(55–60) mm Hg. Due to treatment with sustained-release nifedipine for hypertension, and furosemide and spironolactone for ascites, we cannot be clearly convinced that the blood pressure drop was related to SOF. No other side effects were noted during or after HCV treatment. Although several trials confirmed that SVR12 is an appropriate end point for assessing efficacy of therapy with SOF-containing regimens,^[9,10]^ some individuals experienced relapse between weeks 12 and 24.^[10]^ Monitoring liver function and HCV RNA quantification for 36 weeks, we found that the patient kept in good condition and did not relapse.

## 4 Conclusion

Current HCV treatment guidelines do not offer specific guidance on treatment with DAAs in AHC patients. Therefore, it is particularly important for studies involving IFN-free DAA combinations to further evaluate the role of DAAs in the treatment of AHC. In our patient, HCV infection was eliminated and ALT levels normalized within a 12-week course of SOF and RBV. Thus, with these noteworthy outcomes, this case provides an appropriate reference for other AHC patients with decompensated cirrhosis induced by autoimmune liver disease.
